# DIVERSE WOMEN’S LIVES: THE STUDY OF BIO-PSYCHO-SOCIAL IMPACT ON DISABILITY WITH AGE

**DOI:** 10.1093/geroni/igad104.0681

**Published:** 2023-12-21

**Authors:** Tracie Harrison, Corey Nagel, Tracie Harrison

## Abstract

In this symposium we examine the extent to which psychosocial interventions might promote healthy aging among women with emotional, mobility, and sensory limitations. A growing literature explains the need for culturally tailored care for men and women with disabilities while also stressing the importance of accommodations when living in inaccessible environments. Creating positive social participation amongst diverse groups based on sex/gender with varying levels of function and types of limitations may require more thought and resources than society is willing to forfeit. In this symposium, we begin by examining these issues among people aging in Mexico with few environmental accommodations in place. Creating recognition for the needs of men compared to women who age into mobility limitations in Mexico begins with identifying their unique individual and environmental risks. Next, we examine the needs of women in acute and long-term care with schizophrenia related emotional limitations. Stigma and neglect may create an environmental barrier to healthy aging in the U.S among this population. Our next study examines how sleep duration and quality can impact levels of depression and fatigue for men and women with sensory limitations, drawing attention to the additive effects of emotional changes on functional limitations. Finally, Taylor’s group draws our attention to the work of creating social participation interventions for depression among African American women as they age with severe arthritic pain. We conclude by facilitating a discussion on social and cultural barriers for diverse women aging with functional limitations. This is a Women’s Issues Interest Group Sponsored Symposium.

